# Leiomyomatosis Peritonealis Disseminata during successful pregnancy: First report in Africa

**DOI:** 10.1016/j.ijscr.2025.111025

**Published:** 2025-02-07

**Authors:** Ziyad Fathi AlAtrash, Najwa Eljabu

**Affiliations:** a University of Tripoli, Faculty of Medicine, Tripoli, Libya; b Consultant at Department of Obstetrics and Gynecology, Faculty of Medicine, University of Tripoli, Libya

**Keywords:** Leiomyomatosis Peritonealis Disseminata, Pregnancy, Case report, Histopathology, Libya, Cesarean section, Hormones, Malignancy

## Abstract

**Introduction and importance:**

Leiomyomatosis peritonealis disseminata is a very rare disease that involves the presence of multiple benign tumors of smooth muscle origin all over the peritoneal cavity. Due to its ability to mimic malignant pathologies, it is associated with significant diagnostic and therapeutic challenges. This case highlights a unique case of LPD to further raise awareness and provide useful information on how to manage this condition.

**Case presentation:**

A 28-year-old female was admitted with progressive increase in abdominal distension and discomfort. Imaging studies revealed multiple peritoneal nodules that mimicked metastatic disease. Laparoscopic exploration and histopathological examination subsequently confirmed the diagnosis of LPD. She never had a history of malignancy and hormonal therapy, which are the usual associations with this condition.

**Clinical discussion:**

Diagnosis of LPD is usually missed because its clinically and radiologically smoky appearance of the belly may mimic peritoneal carcinomatosis. Pathogenesis includes hormonal factors, though rare cases are idiopathic. Symptomatic nodules are usually treated with surgical excision and in recurrent cases, attempts at hormonal manipulation can be done.

**Conclusion:**

This case shows the inclusion of LPD in the differential diagnosis of peritoneal masses is of paramount importance, especially in women of childbearing age. Early and accurate diagnosis will help in avoiding the unnecessary aggressive measures.

## Highlights

• First reported case of Leiomyomatosis Peritonealis Disseminata from Libya and Africa, with its international contribution to the literature.
• A challenging diagnosis because of a malignancy-like presentation. Diagnosis, therefore, needs a confirming histopathological examination.
• Histopathologic Diagnosis: Diagnosis confirmed through histopathologic and immuno-histochemical examination showed positive immuno-histochemical reaction for actin and desmin, in contrast to malignancy markers CD34, CD117, DOG1, S100, and β-catenin.
• Successful conservative treatment with good outcomes; therefore, this case provides some hint about the strategies adopted for management. Rare incident to be reported in pregnancy; thus, it opens a scope toward hormonal influence.

## 1 Introduction

Wilson and Peale initially identified a rare benign illness known as leiomyomatosis peritonealis disseminata (LPD) in 1952 [1]. Thereafter, this condition was named by Taubert et al. in 1964 [2]. It typically affects premenopausal women; however, it may also be observed in postmenopausal women. It is characterized by small nodules scattered in the abdominal cavity, mimicking the metastatic behavior originating from smooth muscle cells. The etiological and pathological features of the disease since 1952 remain unclear. The proposed possibilities could be hormonal factors, subperitoneal mesenchymal stem cells, metaplasia, genetics, or iatrogenic post-myomectomy with morcellation [3]. Pathological findings revealed a strong expression of estrogen and progesterone receptors. Recently, with the advancement and widespread use of minimally invasive surgery, the iatrogenic implant theory is regarded as the leading cause; however, the data obtained are merely case reports, and no large studies exist. Additionally, hormonal influences may play a role in the etiology, as explained by cases of hormone-secreting ovarian tumors, patients taking steroid hormones such as COC, and pregnant women with active disease, which cease or degenerate after removing these factors [4,5].

To date, approximately 200 such cases have been reported globally. Because it is a rare clinical condition and due to the absence of typical signs and symptoms, it is frequently misdiagnosed as a metastatic malignancy due to the spread of nodules in the peritoneum. Differential diagnosis includes gastrointestinal stromal tumors, lymphomas, and mesothelioma.

To the best of our knowledge, this is the first reported case in Libya and Africa. We discussed the diagnosis and follow-up of the patient and shared their detailed clinical history. By analyzing the case findings and reviewing the relevant literature, we aimed to enhance the understanding of LPD among clinicians and enrich the existing body of knowledge, as all clinicians should be aware of such potential diagnoses. We obtained consent for publishing the case and used non-identifiable images in our report. This case report conforms to SCARE guidelines [6].

## 2 Case presentation

### 2.1 Medical history

The patient had no history of major medical or surgical illness other than her previous cesarean section. She was a nonsmoker, and she did not use alcohol or contraceptive drugs. She complained of occasional ill-defined abdominal pain without fever, recent infection, or ingestion of oral contrast material. Her menstrual and gynecological history was unremarkable.

The current paper describes the case of a 28-year-old Libyan housewife, admitted on 12/8/2024, for labor pain and vaginal leakage. She is without any connection with chronic disease or history of myomectomy, in her second pregnancy, a G2P1 with previous delivery by cesarean section due to fetal distress, now in week 38 of gestation.

### 2.2 Laboratory tests

Blood reports revealed Hb = 9.9 g/dL, suggesting mild anemia probably due to the physiological alteration of pregnancy. Viral screening was negative for HBsAg, HCV, HIV. Two units of blood were cross matched and arranged. Imaging Studies Ultrasound done preoperatively showed a term pregnancy with cephalic presentation with a normal amount of amniotic fluid. No other abnormalities in abdominal and pelvic organs were noted.

### 2.3 Intraoperative findings

**Fig. 1:**
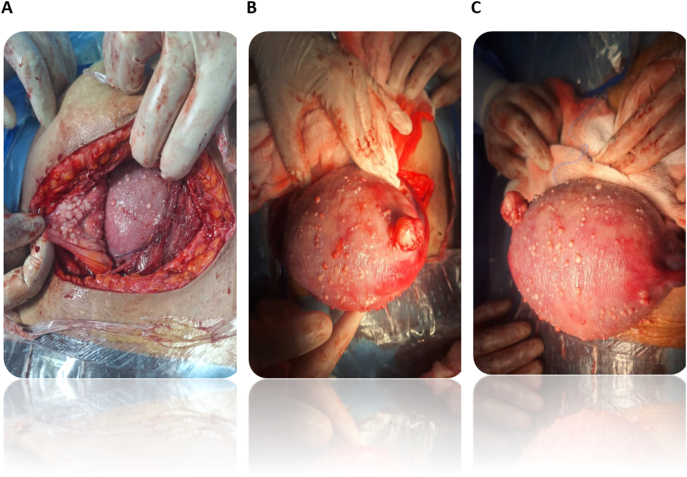
Intraoperative picture shows LPD nodules involving the uterus and peritoneal cavity. Cysts resembling concentrated myomas on the uterine, intestinal, and mesentery surfaces. Sectioned off massive myoma.

Intraoperatively, during LSCS, some whitish nodular lesions measuring 0.5 to 3 cm were seen scattered on the peritoneum, omentum, urinary bladder, and uterus (Fig. 1). Biopsies were taken for histopathology and blood was drawn for CA125 levels-4.36 U/mL. She delivered a live female baby, weighing 3.280 kg, with Apgar scores of 7 at 1 min and 9 at 5 min.

### 2.4 Postoperative course

**Fig. 2:**
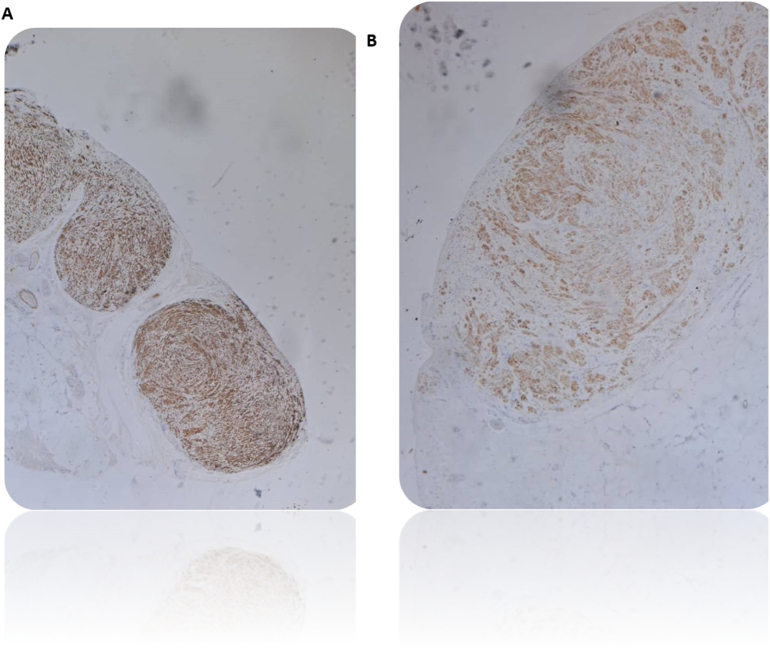
A- Leiomyomatosis peritonealis disseminate (Desmin, 100×): The nodules are strongly positive. B- Leiomyomatosis peritonealis disseminata (SMA, 200×): Positive.

**Fig. 3:**
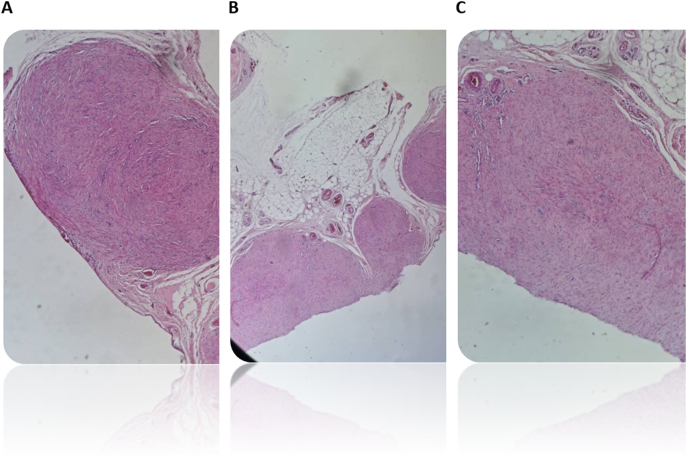
Histopathological biopsy. A&C- Omental biopsy, Leiomyomatosis peritonealis disseminata (H&E,200×): well-defined non capsulated nodules of bland smooth muscle cells. B- Omental biopsy, Leiomyomatosis peritonealis disseminata (H&E, 100x): Multiple well-defined nodules of bland smooth muscle cells.

The patient had an uneventful postoperative recovery; she was ambulatory on day one without complication. She had no significant symptoms except for intermittent vague abdominal pain. Histopathology on 26/8/2024 showed LPD (Fig. 2, Fig. 3). Biopsy negative for nuclear atypia, mitosis, tumor necrosis. Immunohistochemistry: Tumor cells positive for SMA and desmin and negative for CD34, CD117, DOG-1, S100, B-catenin.

### 2.5 Treatment and follow-up

The patient was managed conservatively in the postoperative period, and no medical treatment was instituted immediately in view of the benign nature of the lesions and her aspirations for future fertility. Ultrasound scan abdomen was done revealed normal findings. She was advised about the importance of close follow-up, both imaging-wise and also for hormonal evaluation to monitor recurrence or malignant transformation.

## 3 Discussion

Leiomyomatosis Peritonealis Disseminata (LPD) is an exceptionally rare benign condition typically observed in premenopausal women, although cases have also been reported in postmenopausal women and in association with malignancy [7,8]. To date, less than 200 cases have been documented globally [3]. The preoperative diagnosis of LPD is often challenging because of the asymptomatic nature of the condition. When present, symptoms include abdominal pain, discomfort, vaginal bleeding, and the presence of pelvic masses. The following diagnostic modalities may be helpful: ultrasound, CT, and MRI, although they cannot reliably differentiate LPD from malignant conditions. A definitive diagnosis requires histopathological examination.

The management of LPD is complicated and should be individualized based on factors such as age, symptomatology, fertility desires, and past treatments. Surgical excision is typically reserved for patients who do not desire future fertility and is an attempt to remove all nodules. However, surgery is not void of substantial complications, especially with regard to bowel injury when trying to excise nodules involving the bowel surface. The purpose of medical management is to provide a cure for the disease, particularly for younger women who do not want to lose fertility. Suppression of the disease with anti-estrogen therapies, GnRH antagonists, aromatase inhibitors, or selective progesterone receptor modulators has been performed. The avoidance of postoperative recurrence of the disease is another indication for medical management [[11], [12], [13]].

In the present case, LPD was diagnosed during the second pregnancy. It is likely that the high levels of estrogen and progesterone accompanying pregnancy affect the condition, as both are much higher in pregnant women than in non-pregnant women. While estrogen levels increase by approximately 100-fold compared with non-pregnant women by the third trimester [14], progesterone reaches as high as 200 ng/mL at term [15]. We believe that these hormonal changes might contribute to the development of nodular structures in the abdominal cavity of the patient. Genetic predisposition or the interplay of multiple factors may also contribute to the pathogenesis of this condition. There are no conventional treatment guidelines for this condition; the clinical approach depends on the patient's age, symptoms, fertility requirements, and previous treatments. The value of this report for the scarce literature on the topic consists of pointing out, for the first time, a case from Libya that contributes significantly to the diagnosis and management of LPD in pregnant women. This incidental discovery and lack of symptomatology prior to diagnosis further underline the diagnostic challenge regarding LPD in view of its asymptomatic course and misdiagnosis owing to its malignancy-like presentation. Further histopathological elucidation and planning for long-term management would add strength to this report. Indeed, follow-up of the patient could yield highly valuable information regarding the clinical course of LPD and possible postoperative recurrence. This case emphasizes the need for awareness among clinicians regarding LPD, differential diagnosis from malignancies, and various management options considering future fertility. The authors stress the importance of including LPD in the differential diagnosis of any patient with disseminated intra-abdominal nodules, such as lymphoma, peritoneal carcinomatosis, mesothelioma, metastatic leiomyosarcoma, and gastrointestinal stromal tumors or ovarian tumors. In our case, because of the absence of malignant changes and positive immunohistochemistry profile for (SMA and Desmin) and negative immunohistochemistry profile for (CD34, CD117, DOG-1, S100 and B-cantenin) reliably exclude other differential diagnosis.

## 4 Conclusion

This case report represents a rare, challenging diagnosis of Leiomyomatosis Peritonealis Disseminata that was incidentally diagnosed during a cesarean section. The patient was of reproductive age with no relevant risk factors, and diagnosis was based on the surgical findings and histopathological examination as usual for most cases of LPD. Histopathological examination is the gold standard, and appropriate surgical treatment leads to optimal outcomes. Further research is needed in this area to explore the underlying mechanisms of pathological processes and achieve evidence-based treatment recommendations and the best results and outcomes for patients with LPD. Additionally, studies may offer answers regarding disease progress and the likelihood of recurrence, thus enhancing the existing knowledge and practice.

## Reporting guidelines

This work has been reported in line with the SCARE 2023 criteria^6^[Sohrabi et al., Int J Surg Lond Engl. 2023;109(5):1136].

## CRediT authorship contribution statement

• Study Concept and Design: [Ziyad F Alatrash]
• Data Collection: [Najwa eljabu]
• Data Analysis and Interpretation: [Najwa Eljabu]
• Manuscript Writing: [ZIYAD ALATRASH, NAJWA ELJABU]
• Critical Revision: [ZIYAD ALATRASH]
• Final Approval of the Manuscript: [ZIYAD ALATRASH]

## Consent

Written informed consent was obtained from the patient for publication of this case report and any accompanying images. A copy of the written consent is available for review by the Editor-in-Chief of this journal upon request.

## Ethical approval

Ethical approval was obtained on August 12, 2024.

## Guarantor

The guarantor(s) of this manuscript are [ZIYAD ALATRASH, NAJWA ELJABU], who take full responsibility for the integrity of the study, the accuracy of the data, and the decision to publish the paper.

## Sources of funding

This study received no funding.

## Declaration of competing interest

The authors declare no conflict of interest.
